# Mean arterial pressure at the first prenatal visit as an early predictor of preeclampsia

**DOI:** 10.1002/pmf2.70001

**Published:** 2025-02-27

**Authors:** Ashten B. Waks, Gina Milone, Megan C. Oakes

**Affiliations:** ^1^ Department of Obstetrics and Gynecology MemorialCare Miller Children's and Women's Hospital Long Beach Long Beach California USA; ^2^ Department of Obstetrics and Gynecology, Division of Maternal‐Fetal Medicine University of California Irvine Orange California USA; ^3^ Department of Obstetrics and Gynecology, Division of Maternal‐Fetal Medicine Stony Brook University Stony Brook New York USA

**Keywords:** blood pressure, hypertensive disorders of pregnancy, mean arterial pressure, preeclampsia, preeclampsia prediction, preeclampsia risk screening, preeclampsia risk stratification

## Abstract

**Introduction:**

Preeclampsia is a leading cause of maternal morbidity and mortality worldwide. While screening guidelines exist to identify patients at increased risk of preeclampsia, those currently recommended by the United States Preventive Services Task Force (USPSTF) rely solely on maternal characteristics and are estimated to predict only 15% of eventual cases.

**Objective:**

To determine an early pregnancy mean arterial pressure (MAP) cut‐point for use in preeclampsia risk stratification and to compare the predictive characteristics of this cut‐point with those of the maternal characteristics recommended by the USPSTF screening guidelines.

**Study Design:**

Retrospective cohort study of pregnant persons with a first prenatal visit by 16 weeks’ gestation and delivered in our system from January 2021 to December 2022. MAP was calculated using data captured at the initial visit. Preeclampsia risk was assessed using the 2021 USPSTF risk screening algorithm in which a positive screening was defined as having 1 high or 2 or more moderate risk factors for the condition and a negative screening was defined as having none. Primary outcome was preeclampsia. Youden index was used to identify an optimal early pregnancy MAP cut‐point for predicting preeclampsia. Predictive abilities of this cut‐point and the USPSTF preeclampsia risk screening algorithm were compared using area under the receiver operating characteristic (AUROC) curves, sensitivity, specificity, and positive and negative predictive values.

**Results:**

Of 2169 patients, 230 (11%) developed preeclampsia. The optimal MAP cut‐point for predicting preeclampsia was 88.5 mm Hg. MAP outperformed the USPSTF preeclampsia risk screening algorithm with respect to predicting this diagnosis, as evidenced by AUROC of 0.71 compared to 0.66 (*X* ^2^ = 5.00, *p* = .025). Contrasted with a positive USPSTF risk screening, MAP predicted preeclampsia with slightly improved sensitivity (67 vs. 66%) and negative predictive value (95 vs. 94%), but marginally poorer specificity (65 vs. 67%) and positive predictive value (18 vs. 19%).

**Conclusions:**

Early pregnancy MAP is a modest predictor of preeclampsia and has somewhat improved characteristics as a screening test relative to the USPSTF risk screening algorithm. Moving forward, our early pregnancy MAP threshold may be considered as an adjunct for preeclampsia risk stratification.

## 1 INTRODUCTION

Preeclampsia affects up to 8% of all pregnancies and is a leading cause of maternal morbidity and mortality. In the United States, at least 16% of maternal deaths can be attributed to this diagnosis [1]. Further, estimated expenses related to preeclampsia approach $2.2 billion annually due to increased risks for adverse events for the patient and for preterm birth [2].

Many screening guidelines have been developed to identify patients at increased risk for preeclampsia. The primary benefit of implementing such screening measures is that patients classified as at‐risk can then be treated with low‐dose aspirin, the use of which has been documented to decrease the incidence of preeclampsia by 30% [3]. Currently, the United States Preventive Services Task Force (USPSTF), along with the American College of Obstetricians and Gynecologists (ACOG), recommends universal preeclampsia risk screening for pregnant patients. Their suggested algorithm is based solely on maternal characteristics and relies on a series of “high risk” (e.g., history of preeclampsia, chronic hypertension, pregestational diabetes, pre‐existing kidney disease, autoimmune disease, multifetal gestations) and “moderate risk” (e.g., nulliparity, advanced maternal age, obesity, family history of preeclampsia, in vitro conception, Black race, low income) factors for stratification of preeclampsia risk [4, 5]. This screening tool has the potential to predict approximately 15% of eventual preeclampsia cases depending upon the population in which it is implemented [6].

To improve upon this predictive value, organizations like the Fetal Medicine Foundation have proposed expanded screening tools, which include maternal characteristics in addition to measurements of mean arterial pressure (MAP), uterine artery doppler pulsatility, serum placental growth factor (PlGF), and pregnancy‐associated plasma protein A (PAPP‐A) [7]. These algorithms capture closer to 75% of patients at risk for preeclampsia but are also associated with increased costs due to the need for supplementary ultrasound and laboratory studies that are not yet standard of care in the United States [7].

To retain this improved accuracy while decreasing screening costs, a small number of studies have assessed the benefit of MAP alone as an adjunct to maternal characteristics, as this measurement is already obtained through blood pressure surveillance at prenatal visits [8, 9, 10, 11, 12]. Such studies have demonstrated improved predictive value when MAP is added to existing algorithms, with detection rates as high as 72% [8] to 76% [10]. Although these studies propose that MAP between 85 and 95 mmHg is associated with an increased risk for preeclampsia, there is no identified optimal cut‐point [8, 9, 10, 11, 12]. Elucidating such a value might allow us to better capture patients who are missed with existing screening algorithms.

Accordingly, we designed a study to determine an early pregnancy MAP cut‐point for use in preeclampsia risk stratification and to compare the predictive abilities of this cut‐point with those of the maternal characteristics recommended by the USPSTF. We hypothesized that the predictive value of MAP at the first prenatal visit would be non‐inferior to that of the USPSTF screening algorithm.

## 2 MATERIALS AND METHODS

We conducted a retrospective cohort study at MemorialCare Miller Children's and Women's Hospital Long Beach and MemorialCare Saddleback Medical Center, both tertiary care centers serving diverse metropolitan areas. The study was approved by the Memorial Health Services (MHS) Institutional Review Board. Electronic medical records (EMRs) were obtained for pregnant patients within the MemorialCare system who delivered beyond 20 0/7 weeks’ gestation from January 1, 2021 through December 31, 2022. These dates were selected to coincide with implementation of the revised USPSTF screening algorithm introduced in 2021 [4].

Records were then reviewed, and patients with a documented prenatal care visit within our health system before 16 0/7 weeks’ gestation were included in the study cohort. Patients with no documented prenatal care visit within our health system or a first visit after 16 0/7 week's gestation were excluded. This gestational age limit was established to account for the optimal gestational age by which low‐dose aspirin would be initiated, as this is the intended outcome of positive preeclampsia risk screening [4]. Of note, all patients receiving prenatal care in health system clinics and then delivering at either of the study centers were privately insured at the time of establishing care.

### 2.1 Data collection

For eligible patients, systolic and diastolic blood pressures at the initial prenatal visit were abstracted from the EMR. These values were used to calculate a patient‐specific MAP according to Gauer's method ([systolic blood pressure + (2 × diastolic blood pressure)]/3) [13]. In addition, data on the socioeconomic, obstetric, and medical characteristics in the USPSTF risk screening algorithm were collected. This included maternal age at delivery, gravidity, parity, self‐identified race/ethnicity, body mass index (BMI), history of preeclampsia in a previous pregnancy, and diagnosis of comorbid conditions (e.g., chronic hypertension, pregestational diabetes, renal disease, autoimmune disease) [3]. Based on unreliable reporting in the EMR, data on income, family history of preeclampsia, and a history of adverse pregnancy outcomes—all of which are classified as “moderate risk” factors in the USPSTF screening algorithm—could not be obtained.

Using the available data, the USPSTF risk screening algorithm was subsequently applied to each patient. Patients were defined as having a positive screening if they had at least one “high risk” or two or more “moderate risk” factors for preeclampsia. Patients were defined as having a negative screening if they had only one “moderate risk” or no identifiable risk factors for preeclampsia [4, 5]. Of note, the USPSTF currently acknowledges that the “moderate risk” factors of self‐identified Black race and low income may be sufficient to warrant low‐dose aspirin initiation even in the absence of other risk factors [4, 5]. However, as there was overlap in the timing in which this clarification was published and our study was conducted, this practice had not yet been widely adopted in our health system. Accordingly, these risk factors were still considered as one of two required “moderate risk” factors in our application of the USPSTF guidance.

### 2.2 Outcomes

The primary outcome was the diagnosis of preeclampsia. The occurrence of this outcome was confirmed using one of three EMR‐based strategies: (1) Provider documentation of a diagnosis of preeclampsia after 20 weeks’ gestation, (2) identification of a combination of elevated blood pressures and abnormal serum or urine laboratory data, as defined by ACOG [1], or (3) capture of relevant ICD‐10 codes (O14.0 mild to moderate preeclampsia, O14.1 severe preeclampsia, O14.2 HELLP syndrome, O14.9 unspecified preeclampsia) [14]. The secondary outcomes were diagnosis of preeclampsia with severe features [14], preterm preeclampsia (diagnosis before 37 0/7 weeks’ gestation), and early preterm preeclampsia (diagnosis before 34 0/7 weeks’ gestation).

### 2.3 Statistical analysis

Demographic and clinical characteristics were compared between patients with and without preeclampsia using Student's *t*‐test for continuous variables and chi‐square or Fisher's exact test for categorical variables. The normality of distribution for continuous variables was evaluated using Kolmogorov‐Smirnov test.

Area under the receiver operating characteristic (AUROC) curves were constructed to assess the predictive ability of early pregnancy MAP for the eventual diagnosis of preeclampsia as well as for application of the USPSTF risk screening algorithm in our population. These AUROC curves were compared based on the method outlined by Delong and colleagues [16]. An AUROC curve greater than 0.7 was taken to represent an accurate model. The optimal early pregnancy MAP cut‐point for predicting a later diagnosis of preeclampsia was then determined using the Youden index, which maximizes sensitivity and specificity [15]. Predictive statistics for the optimal early pregnancy MAP cut‐point and application of the USPSTF risk screening algorithm were also compared, including sensitivity, specificity, positive and negative predictive values, and positive and negative likelihood ratios.

A subgroup analysis was planned to evaluate the optimal early pregnancy MAP cut‐point for predicting preeclampsia and the predictive characteristics of this cut‐point among patients who screen negative on the USPSTF risk screening algorithm. Our reason for conducting this subgroup analysis was to assess if patients without recognized demographic risk factors for preeclampsia may still have identifiable clinical risk factors which may benefit from management with prophylactic low‐dose aspirin [3, 4].

Sample size was not estimated *a priori*, as all patients meeting inclusion criteria during the study period were included. Analyses were completed using STATA version 18 (College Station, TX), and a two‐sided *p*‐value of 0.05 was considered significant.

## 3 RESULTS

**FIGURE 1:**
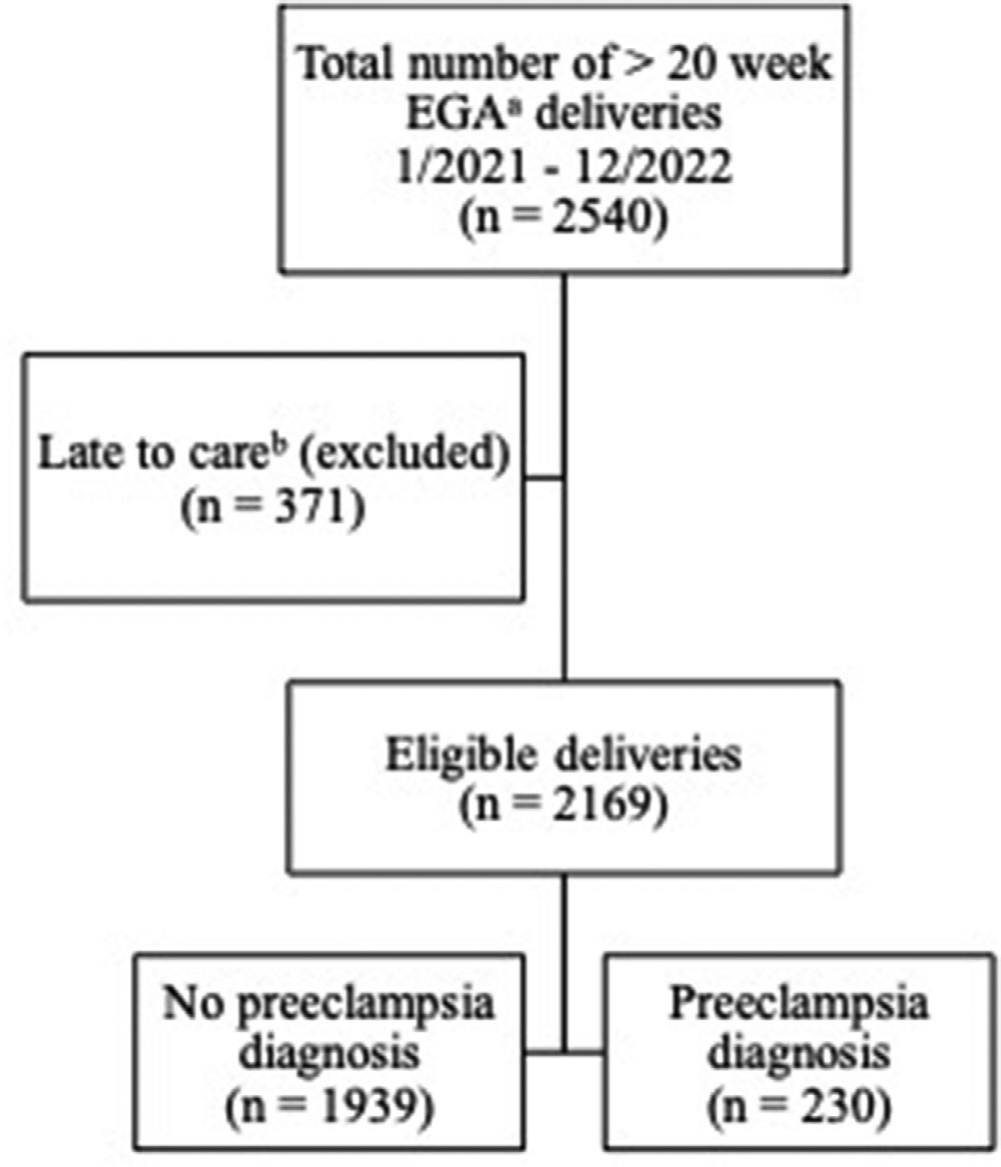
Flow diagram for derivation of the study population. ^a^Estimated gestational age. ^b^First prenatal care visited documented after 16 weeks’ gestation.

A total of 2540 deliveries at 20 0/7 weeks’ gestation or beyond occurred among health system patients during the study period (Figure 1). Of these, 2169 (85%) were to health system patients with a documented prenatal care visit before 16 0/7 weeks’ gestation and were included in the study cohort. The remaining 371 (15%) deliveries, which were to health system patients with a first prenatal care visit after 16 0/7 week's gestation, were excluded. The primary outcome of preeclampsia was diagnosed in 230 (10.6%) eligible patients. Among patients with preeclampsia, 114 (50%) cases were further diagnosed with severe features, 64 (28%) with preterm preeclampsia, and 15 (7%) with early preterm preeclampsia.

Baseline characteristics for the entire cohort and compared between cohorts with and without a diagnosis of preeclampsia are presented in Table 1. The mean maternal age at delivery for the study cohort was 32.8 years. The majority of patients were white (42.7%), though large Asian (25%) and Hispanic (22%) populations were included in the cohort as well. Patients with and without preeclampsia were similar with respect to maternal age at delivery, race/ethnicity, incidence of multifetal gestations, use of assisted reproductive technologies, and diagnoses of pre‐existing renal and autoimmune disease. As anticipated, patients with an eventual diagnosis of preeclampsia were significantly more likely to be obese (45% vs. 21%, *p* < 0.001) and nulliparous (64% vs. 53%, *p* = 0.001). Patients with an eventual diagnosis of preeclampsia were also significantly more likely to have chronic hypertension (21% vs. 4%, *p* < 0.001) and pregestational diabetes (4% vs. 1%, *p* = 0.003). Regarding delivery characteristics, patients with preeclampsia were also more likely to deliver via cesarean (41% vs. 30%, *p* = 0.003), at an earlier gestational age (38 vs. 39 weeks, *p* < 0.001), and to have neonates with a lower birthweight (3125 vs. 3322 g, *p* < 0.001).

**TABLE 1:** Baseline characteristics of the study population.

	Number (%)^a^		
Variable	Entire cohort (*n* = 1325)	Preeclampsia diagnosis (*n* = 146)	No preeclampsia diagnosis (*n* = 1179)
**Maternal age**, years, Mean (SD)	32.8 (4.6)	33.2 (4.8)	32.7 (4.6)
**Race or ethnic status**			
White	925 (42.7)	89 (38.7)	836 (43.1)
Hispanic	486 (22.4)	61 (26.5)	425 (21.9)
Black	67 (3.1)	12 (5.2)	55 (2.8)
Asian	535 (24.7)	50 (21.7)	485 (25.0)
Other, unknown	156 (7.2)	18 (7.8)	138 (7.1)
**Obesity (BMI > 30 kg/m^2^)**	504 (23.2)	103 (44.8)	401 (20.7)
**Nulliparity**	1162 (53.7)	147 (63.9)	1015 (52.5)
**Multifetal gestation**	36 (1.7)	5 (2.2)	31 (1.6)
**IVF conception**	126 (5.8)	16 (7.0)	110 (5.7)
**Obstetric co‐morbidities**			
Chronic hypertension	132 (6.1)	49 (21.3)	83 (4.3)
Pregestational diabetes	25 (1.2)	8 (3.5)	17 (0.9)
Pregestational renal disease	5 (0.2)	1 (0.4)	4 (0.2)
Autoimmune disease	25 (1.2)	5 (2.2)	20 (1.0)
**Low‐dose aspirin use in pregnancy**	439 (20.3)	112 (48.7)	327 (16.9)
**MAP**, mm Hg, Median (IQR)	86 (80‐92)	92 (87–99)	85 (79–91)
**Mode of delivery**			
Spontaneous vaginal	1287 (59.3)	117 (50.9)	1170 (60.3)
Operative vaginal	200 (9.2)	18 (7.8)	182 (9.4)
Cesarean	682 (31.4)	95 (41.3)	587 (30.3)
**Gestational age**, weeks, Median (IQR)	39 (38‐40)	38 (37‐39)	39 (38‐40)
**Birthweight**, grams, Median (IQR)	3305 (2995‐3610)	3125 (2730‐3460)	3322 (3035‐3629)

Abbreviations: BMI, body mass index; IQR; interquartile range; IVF, in vitro fertilization; MAP, mean arterial pressure.

^a^ Except where otherwise noted.

**FIGURE 2:**
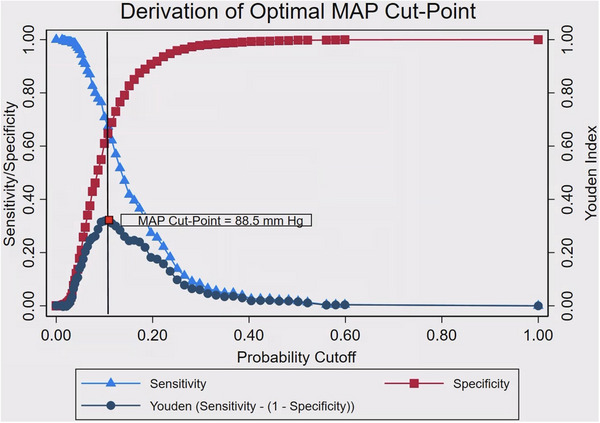
Derivation of an optimal mean arterial pressure (MAP) cut‐point according to the Youden index.

The median MAP at the first prenatal visit was 86 mm Hg (IQR 80–92) across the entire study cohort, 92 mm Hg (IQR 87–99) in patients with preeclampsia, and 85 mm Hg (IQR 79–91) in patients without preeclampsia (*p* < 0.001). The optimal early pregnancy MAP cut‐point for predicting preeclampsia was 88.5 mm Hg (Figure 2). As demonstrated in Table 2, this early pregnancy MAP cut‐point predicted preeclampsia with a sensitivity of 67% (95% CI 60.9–73.4), specificity of 65% (95% CI 62.6–66.9), positive predictive value of 18% (95% CI 16.0–19.1), and negative predictive value of 95% (95% CI 93.7–95.6). Of note, although the optimal early pregnancy MAP cut‐point for predicting preeclampsia differed slightly among patients with preeclampsia with severe features (86.5 mm Hg), preterm preeclampsia (88.5 mm Hg), and early preterm preeclampsia (86.5 mm Hg), there were no meaningful differences in the predictive characteristics associated with these cut‐points.

**TABLE 2:** Predictive characteristics of “optimal cut‐points” of mean arterial pressure for predicting preeclampsia.

Characteristic	Preeclampsia, any EGA (*n* = 230)	Preeclampsia, severe (*n* = 114)	Preeclampsia, < 37w0d (*n* = 64)	Preeclampsia, < 34w0d (*n* = 15)
**Optimal cut‐point**, mm Hg	88.5	86.5	88.5	86.5
Area under ROC curve (95% CI)	0.71 (0.67–0.74)	0.64 (0.62–0.71)	0.70 (0.62 ‐0.78)	0.73 (0.63–0.86)
Sensitivity (95% CI)	67.4% (60.9–73.4)	74.6% (65.6–82.3)	78.1% (66.0–87.5)	99.3% (68.1–99.8)
Specificity (95% CI)	64.7% (62.6–66.9)	53.0% (50.9–55.2)	62.4% (60.3–64.5)	51.9% (49.8–54.0)
Positive predictive value (95% CI)	17.5% (16.0–19.1)	15.0% (13.6–16.6)	6.0% (5.3–6.9)	1.4% (1.2–1.6)
Negative predictive value (95% CI)	94.7% (93.7–95.6)	94.9% (93.2–96.3)	98.9% (98.2–99.3)	99.9% (99.4–99.9)
Positive likelihood ratio (95% CI)	1.91 (1.71–2.13)	1.59 (1.41–1.78)	2.08 (1.81–2.39)	1.94 (1.68–2.24)
Negative likelihood ratio (95% CI)	0.50 (0.42–0.61)	0.48 (0.35–0.66)	0.35 (0.22–0.56)	0.13 (0.02–0.85)

**FIGURE 3:**
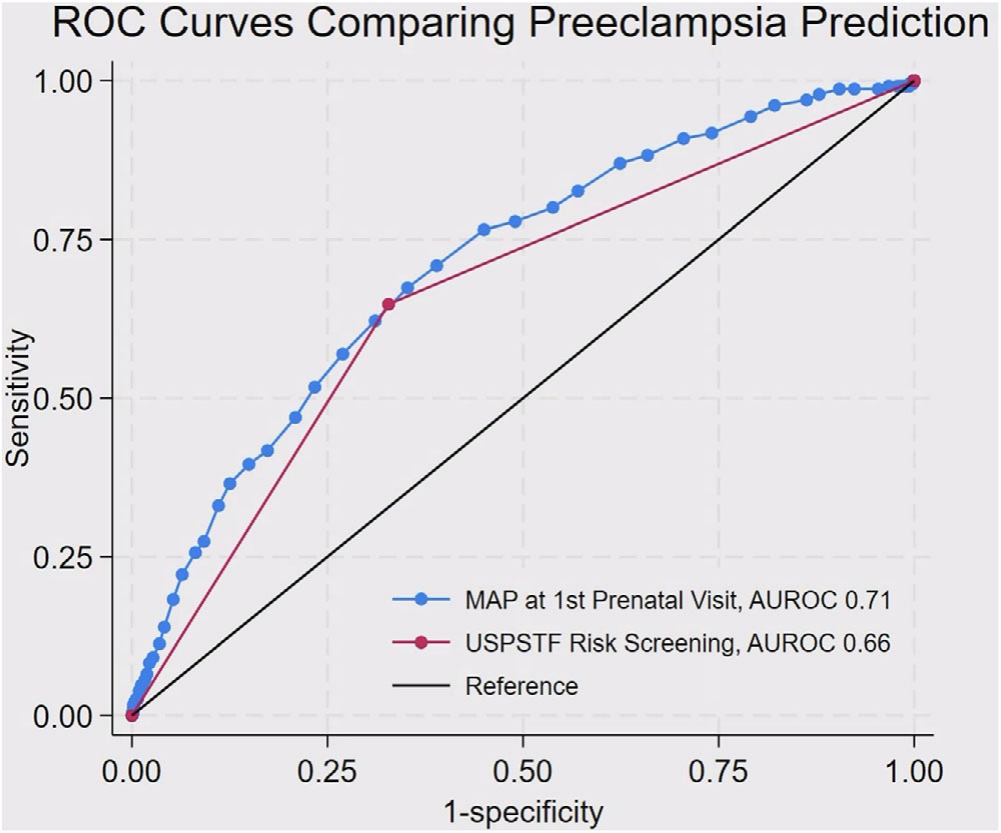
Receiver operating characteristics (ROC) curve comparing mean arterial pressure (MAP) to the application of the United States Preventive Services Task Force (USPSTF) preeclampsia risk screening algorithm for predicting preeclampsia. AUROC, area under the receiver operating characteristics curve.

Early pregnancy MAP was modestly predictive of an eventual preeclampsia diagnosis, with an AUROC of 0.71 (95% CI 0.67–0.74). In comparison to early pregnancy MAP, application of the USPSTF risk screening algorithm in our population underperformed in its ability to predict preeclampsia (AUROC 0.71 vs. 0.66, *X* ^2^ = 5.00, *p* = .025) (Figure 3). Furthermore, as shown in Table 3, application of the USPSTF risk screening algorithm in our population had somewhat poorer sensitivity (66%, 95% CI 59.1–71.8) and negative predictive value (94%, 95% CI 93.2–95.2) relative to use of an early pregnancy MAP cut‐point. Otherwise, application of the USPSTF risk screening algorithm in our population had marginally improved specificity (67%, 95% CI 64.7–68.9) and positive predictive value (19%, 95% CI 17.3–20.8) relative to use of an early pregnancy MAP cut‐point.

**TABLE 3:** Predictive characteristics of USPSTF risk categories for predicting preeclampsia.

Characteristic	USPSTF Risk Categories
Area under ROC curve (95% CI)	0.66 (0.63–0.69)
Sensitivity (95% CI)	65.7% (59.1–71.8)
Specificity (95% CI)	66.8% (64.7–68.9)
Positive predictive value (95% CI)	19.0% (17.3–20.8)
Negative predictive value (95% CI)	94.3% (93.2–95.2)
Positive likelihood ratio (95% CI)	1.98 (1.77–2.21)
Negative likelihood ratio (95% CI)	0.51 (0.43–0.62)

Finally, the results of our subgroup analysis for patients who screened negative on the USPSTF risk screening algorithm are presented in Table 4. The optimal early pregnancy MAP cut‐point for predicting preeclampsia in this population was identical to that in the broader study cohort (88.5 mm Hg). However, with an AUROC of only 0.63, this optimal early pregnancy MAP cut‐point was not predictive of preeclampsia among patients with a negative result on the USPSTF risk screening algorithm. The poor predictive abilities of early pregnancy MAP among patients in this sub‐group were consistent for the secondary outcomes of preeclampsia with severe features and preterm preeclampsia as well.

**TABLE 4:** Predictive characteristics of “optimal cut‐points” of mean arterial pressure for predicting preeclampsia among patients without an indication for antenatal aspirin.

Characteristic	Preeclampsia, any EGA (*n* = 79)	Preeclampsia, severe (*n* = 37)	Preeclampsia, < 37w0d (*n* = 14)	Preeclampsia, < 34w0d (*n* = 4)
**Optimal cut‐point**, mm Hg	88.5	83.5	86.5	94.5
Area under ROC curve (95% CI)	0.63 (0.60–0.72)	0.58 (0.52–0.69)	0.77 (0.71–0.88)	0.69 (0.35–0.97)
Sensitivity (95% CI)	55.7% (44.1–66.9)	67.6% (50.2–82.0)	92.9% (66.1–99.8)	50.0% (6.8–93.2)
Specificity (95% CI)	71.1% (68.5–73.5)	47.9% (45.3–50.7)	60.5% (57.9–63.1)	88.6% (86.8–90.2)
Positive predictive value (95% CI)	10.5% (8.7–12.7)	3.5% (2.8–4.3)	2.4% (2.0–2.8)	1.26% (0.5–3.3)
Negative predictive value (95% CI)	96.3% (95.5–97.1)	98.2% (97.2–98.8)	99.9% (99.2–99.9)	99.8% (99.6–99.9)
Positive likelihood ratio (95% CI)	1.92 (1.55–2.39)	1.30 (1.03–1.63)	2.35 (2.00–2.76)	4.37 (1.62–11.77)
Negative likelihood ratio (95% CI)	0.62 (0.49–0.80)	0.68 (0.42–1.08)	0.12 (0.02–0.78)	0.56 (0.21–1.50)

## 4 DISCUSSION

We identified an optimal early‐pregnancy MAP cut‐point of 88.5 mm Hg for use in preeclampsia risk evaluation among 2169 patients whose deliveries coincided with implementation of the 2021 updates to the USPSTF preeclampsia risk screening algorithm. As hypothesized, the predictive characteristics of this optimal early‐pregnancy MAP cut‐point were not inferior to those of the USPSTF risk screening algorithm in our patient population. In fact, the optimal early pregnancy MAP cut‐point had a superior AUROC, sensitivity, and negative predictive value compared to application of the USPSTF risk screening algorithm.

Previous studies interrogating the relationship between early pregnancy MAP and preeclampsia selected MAP thresholds without clear guidance from the existing literature [9, 12, 17]. For example, Suksai et al. calculated MAP based on blood pressure at the first prenatal visit for over 4600 patients and found that mean MAP was 88.7 mm Hg in those who developed preeclampsia and 82.3 mm Hg in those that did not (*p* < 0.001) [12]. However, they then chose a MAP threshold of greater than or equal to 95 mm Hg for inclusion in predictive modeling, which they determined was associated with a 2.6‐fold increased odds of developing preeclampsia [12]. Similarly, Miller and colleagues averaged blood pressures across the first trimester to compute an early pregnancy MAP for close to 2000 patients and then placed patients into pre‐specified MAP categories of less than 79 mm Hg, 79–83 mm Hg, 84–88 mm Hg, and greater than 89 mm Hg [17]. Their results demonstrated that MAP in the highest quartile was associated with a 3‐times increased risk of eventual preeclampsia [17]. An additional study by Gasse et al. evaluated first trimester MAP in terms of multiples of the median (MoM) for nearly 4800 patients [9]. These authors did not publish mean MAP data or establish thresholds for their MoM values *a priori*, though concluded that MAP with a MoM between 1.0 and 1.2 was associated with an increased risk of preeclampsia [9]. While each of these studies assessed MAP in distinct ways and none computed an optimal MAP cut‐point according to the Youden index, when they examined the predictive characteristics of MAP, the results were similar. Specifically, like our study, the aforementioned studies found that early pregnancy MAP was only slightly predictive of preeclampsia, with AUROC ranging from 0.71 to 0.77 [9, 12, 17].

In spite of the modest predictive characteristics of MAP in relation to the diagnosis of preeclampsia, MAP may still play a meaningful role in risk stratification. In 2017, the American College of Cardiology (ACC) and American Heart Association (AHA) introduced new blood pressure categories for use in adult patients. They now recommend a diagnosis of pre‐hypertension for patients with systolic blood pressures between 120 and 129 mm Hg, stage 1 hypertension for patients with blood pressures between 130–139/80–89 mm Hg, and stage 2 hypertension for patients with blood pressures above 140/90 mm Hg [18]. While the obstetrics community is engaged in ongoing discussions and clinical research regarding how to best incorporate the ACC/AHA's diagnostic criteria into practice, many prenatal care providers continue to use blood pressures above 140/90 mm Hg to diagnose hypertension [1]. Use of this parameter may result in missed opportunities to more carefully surveille or even prescribe low‐dose aspirin to patients who would otherwise be diagnosed with pre‐ or stage 1 hypertension outside pregnancy.

We propose that assessment of MAP at the first prenatal visit may help to fill this gap until there is further consensus between the ACC/AHA and obstetric providers in terms of how to capture patients with abnormal blood pressure during pregnancy. This might involve treating our optimal early pregnancy MAP cut‐point as a “moderate risk” factor in the USPSTF preeclampsia risk screening algorithm. Alternatively, it might also involve use of MAP categories such as those suggested by Melgarejo and colleagues. Their MAP classification system submits that MAP less than 90 mm Hg corresponds to normotension, 90–91.9 mm Hg to prehypertension, 92–95.9 mm Hg to stage 1 hypertension, and > 96 mm Hg to stage 2 hypertension [19]. As our optimal early pregnancy MAP threshold was within the upper limit of the normotensive category, any patient in the pre‐, stage 1, or stage 2 hypertension MAP classes should likely be considered at risk for preeclampsia.

Our study was strengthened by execution of a novel design across a cohort of patients similar in size to that of other studies on MAP as a predictor of preeclampsia [9, 12, 17]. As the patients who receive prenatal care with and deliver in our health system are considered “low risk,” we were initially concerned that use of this population may result in a low incidence of our study outcomes and, thus, limit our abilities to generate clinically meaningful results. In reality, the incidence of common obstetrical comorbidities in the study sample—which can be used as a proxy for baseline risk—was similar to that in the United States as a whole. For example, 6.1% of the cohort had chronic hypertension compared to up to 5% in the general population [20], and 1.2% of the cohort had pre‐gestational diabetes compared to 1%–2% in the general population [21]. Moreover, the preeclampsia incidence of nearly 11% in our sample exceeds that of the general population (8%), further highlighting the adequacy of this cohort for meeting our objectives.

At the same time, because the obstetric practices within this health system do not currently accept Medicaid insurance, all patients included in our analysis were privately insured. This suggests that there was likely underrepresentation of patients with low income or socioeconomic status—a “moderate risk” factor for preeclampsia [3, 4]—in our population. United States birth certificate data suggests that low socioeconomic status is actually the most common preeclampsia risk factor, affecting nearly 47% of all patients, which may also limit our study's generalizability [22]. Another potential limitation of our study is reliance on EMR documentation and coding to ascertain predictors and outcomes for our models. Specifically, there was inconsistent documentation of family history of preeclampsia and certain adverse pregnancy outcomes (e.g., low birthweight), both “moderate risk” factors for preeclampsia. For this reason, we withheld these characteristics from our application of the USPSTF risk screening algorithm, which may have led to underestimation of the true number of patients with a positive screening. Finally, patients included in our cohort delivered as early as January 2021, while the USPSTF preeclampsia risk screening algorithm applied in this study was not formally published until September of that year [4]. For the purposes of this study, the algorithm was applied retrospectively by research staff after all included patients had delivered, so this timeline was unlikely to have meaningfully impacted the findings.

## 5 CONCLUSIONS

In conclusion, results from this retrospective cohort study show that, while early pregnancy MAP may be superior to the USPSTF preeclampsia risk screening algorithm for identifying patients at risk for this condition, MAP alone is only modestly predictive of preeclampsia. Even so, elucidation of an early pregnancy MAP cut‐point that can be used for preeclampsia risk stratification may still enhance our ability to identify at‐risk patients. Before the early pregnancy MAP cut‐point calculated in this study can be introduced into clinical practice, though, future studies would be required to validate this metric both alone and in conjunction with other preeclampsia risk screening modalities.

## CONFLICT OF INTEREST STATEMENT

The authors declare no conflicts of interest.

## ETHICS STATEMENT

The study was approved by the Memorial Health Services (MHS) Institutional Review Board.

## Data Availability

The data that support the findings of this study are available from the corresponding author, Ashten Waks, upon reasonable request.
